# Wellness Centre: An Evidence-Guided Approach to Delivering Culturally Relevant Community Psychogeriatric Services for Chinese Elders

**DOI:** 10.5402/2012/815707

**Published:** 2012-03-01

**Authors:** Kar C. Chan, Joel Sadavoy

**Affiliations:** ^1^Wellness Centre, Department of Psychiatry, Mount Sinai Hospital, Toronto, ON, Canada M5T 3L9; ^2^Department of Psychiatry, University of Toronto, Toronto, ON, Canada M5S 1A1; ^3^Applied General Psychiatry, Community Psychiatry and Geriatric Psychiatry Programs, Mount Sinai Hospital, Toronto, ON, Canada M5T 3L9

## Abstract

Ethnic elders are commonly reluctant to access mental health services and their mental health problems are often overlooked and detected late in the course of illness. Prior studies identified major barriers to ethnic seniors accessing appropriate mental health care demonstrating that language and cultural beliefs cannot be ignored if effective mental health services are to be provided to patients from diverse cultural groups. These are particularly important when care is needed by less acculturated immigrant ethnic seniors for whom language barriers are often greatest. Differences in conceptions of mental distress affect ethnic seniors' choice of help-seeking and often discourage or divert aged persons from utilizing mainstream conventional psychiatric care. Despite the extensive need for appropriate service models for ethnic populations, there have been limited data and models to illustrate how these programs can be systematically and effectively integrated within the mainstream mental health service framework. This paper describes an innovative, mainstream, community-based psychogeriatric service delivery model developed for Chinese seniors in Toronto, Canada, aiming at improving their access to care and enhancing earlier mental health problem detection. The important concepts and strategies of designing and operating a culturally acceptable program are illustrated supported by program data and the challenges analyzed.

## 1. Introduction

Canada's visible-minority population including its geriatric age group is growing at a much faster rate than the general population. Between 2001 and 2006 it grew by 27% [1]. It is estimated that by 2017, one in five Canadians will be a visible minority, and half of them will be Chinese and South Asian [2]. This ethnic diversity is particularly notable in large urban centers such as Toronto and Vancouver.

North American studies have consistently revealed a high prevalence of mental disorders among Chinese elders compared to their general population counterparts but their mental health problems are often underdiagnosed and undertreated [3–5]. Barriers to care are largely explained by eight key factors: stigma [6]; poor knowledge of resources [7]; reliance on family's support for care-seeking and tendency of families to contain problems until they turn into crisis [7]; lack of linguistic and culturally appropriate service [2, 7, 8]; worries over medication side effects and the dominance of drug therapy [8]; underdetection at the primary care level [9, 10]; geographical inaccessibility of services [7, 11]; challenges in navigating the complex health care system and dealing with long waiting lists [8].

Culturally determined conceptions of mental health and illness determine help-seeking behaviors, shaping treatment preferences, diverting or postponing receipt of appropriate mainstream care, influencing satisfaction with services, and ultimately reducing compliance and willingness to continue treatment.

To make services acceptable and effective, mental health service planners and providers must address the identified service barriers and take into consideration the unique cultural values and expectations of ethnic immigrant populations [12–14].

## 2. Theoretical Model for the Wellness Centre

The Wellness Centre, a specialized mental health service for older Chinese adults, was developed to provide innovative and systematic solutions to the established barriers as well as address cultural factors that affect the efficacy and efficiency of mental health services. The centre's structure and function are based on a core philosophy that an ethnocultural community mental health program will most effectively attract and engage those in need only when the program meets the cultural and linguistic needs of the specific target group. The effectiveness of engagement also depends heavily on whether the mix of medical and wellness services offered match the expectations and needs of clients who are at specific points on the health-illness continuum as well as their culturally derived concepts of the determinants of mental health. These concepts include the following: mental health is intrinsically connected to physical health [8]; mental health may be maintained or restored through diet, exercise, and bodily interventions [15]; self-help and body-mind alternatives foster better mental health and minimize stigma [6, 16].

The innovation of this psychogeriatric centre is that it delivers geriatric mental health services based upon a seamlessly integrated traditional and western-medical-based philosophy. It formally addresses culture-specific determinants of mental health, supports the family-as-the-unit-of-care concept, and encourages wellness principles that enhance clients' active involvement in their own health decision-making, health promotion, and self-monitoring while integrating culturally preferred wellness options with evidence-based western treatments.

**Figure 1:**
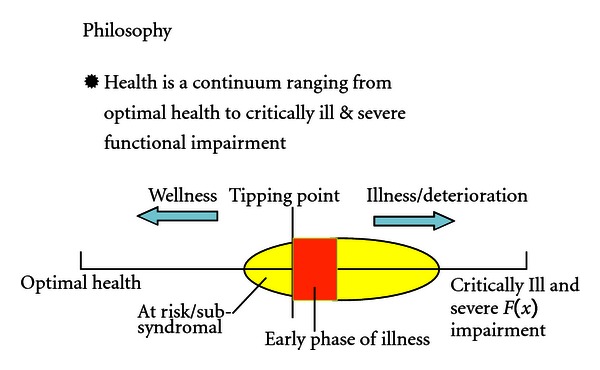
Conceptual model of health continuum.

The wellness-based philosophy of the centre is an expansion of the “healthy/dis-ease”continuum model which more commonly appears in health promotion literature [17, 18] (Figure 1).

In this paradigm, an individual's health condition is a dynamic as opposed to static point on the continuum. Hence, at different points individuals have to employ different combinations of health, medical, and wellness strategies. Initially these include actively engaging in self-help and prevention strategies to advance or maintain their own health, as well as to prevent illness or health deterioration [19].

The tipping point, a concept introduced into the model, is the watershed at which an individual has displayed clear and diagnosable symptoms of an illness/disorder and is no longer able to maintain daily functioning levels. Beyond this point, individuals are less able to rely merely on their own self-help strategies to restore their health condition. However, Chinese elders in particular do not always discriminate well the point at which they “tip” [7, 8] perhaps attributable to a combination of the lack of concomitant endorsement from family, reliance on personal endurance to overcome challenges—a manifestation of the unique “locus of control” beliefs that are common among less acculturated Asians [20], and culturally determined views of mental distress that do not equate it with a condition requiring professional intervention [15, 21]. Hence it is only when an unmanageable crisis arises that urgent western health care is finally sought leading to delayed intervention and increased need for emergency/crisis care [22]. The Wellness Centre is designed to encourage early identification and intervention and avoid crises.

## 3. The Wellness Centre Clinical Program

Clients who are either self or professionally referred are comprehensively assessed. In addition to a full psychiatric workup, illness experience and other culturally important factors [23] are assessed concurrently. This involves eliciting the patient's own illness explanation, an appraisal of their culture and wellness orientation and their belief in the impact of these factors on their illness, symptom presentation, coping, treatment preference, and traditional and mainstream pattern of help-seeking.

Culturally relevant wellness program options are always discussed and integrated as adjunctive to western psychiatric treatments. Traditional wellness interventions may be offered alone temporarily in the initial phases of intervention when clients are resistant to medical interventions. Participating in wellness interventions helps these clients to maintain regular contact and interact with the centre's program and clinical staff who can provide continuing support and education for them to gain trust in and knowledge of their illness and recommended treatment. These include traditional exercises (e.g., Tai-chi and Qi-gong groups), pain management, holistic health education, teaching of relaxation and stress reduction methods, and dietary consultation.

### 3.1. The Wellness Centre's Evidence-Guided Systematic Approach to Addressing Barriers to Access to Mental Health Care

The following strategies were instituted by the Wellness Centre in response to each identified barrier highlighted in the introduction.

#### 3.1.1. The Stigma of Psychiatric Illness

Hinshaw [24] has suggested that there are several key elements to destigmatize mental health services: accessibility, changing views, improving media portrayals and sensitivity to mental illness, and policy initiatives. From its inception the Wellness Centre has integrated these elements into its service approach and delivery.

The physical design of the centre incorporates culturally familiar elements, such as decorations, colours, and signage, which promote a sense of acceptance and accessibility among clients and visitors. The reception area is designed to look like a drop-in centre rather than a conventional mental health out-patient clinic. Wellness activities are open to patients, their families, and nonpatients alike. In some group activities there is no formal differentiation between “patients” and others who are coming solely for the “wellness” component which helps to normalize and de-stigmatize the experience of receiving care. Stigma is further addressed through allocating time and program resources for community outreach and mental health education which are often delivered through ethnic media channels. The centre is also proactively involved in professional education, system-wide discussion, and planning to enhance and advocate for culturally relevant antistigma initiatives and care model development.

#### 3.1.2. Poor Knowledge of Available Resources

The Wellness Centre assiduously promotes partnerships and service collaborations with prominent ethnic community agencies to expand its outreach and services to the target population. It advertises its services and related support resources widely, utilizing language appropriate community mental health education, onsite wellness programs, bilingual information packages and program brochures, and the sharing of information through community service networks.

#### 3.1.3. The Central Importance of Family in the Care-Seeking Process of Chinese Seniors and the Delay in Service Access Caused by Attempts to Contain Problems within the Family

Chinese cultural norms place a moral obligation on families to offer support to a member in distress. Consequently, family is often the first and most important resource that a Chinese senior will turn to for support and help [25]. Family-based illness appraisal [26] will often determine whether the family accepts that the senior has a problem and engages in appropriate help-seeking.

However, families often misinterpret or ignore symptoms of psychogeriatric disorders [27]. Therefore, the centre devotes a major part of its educational outreach to modifying the mental health appraisal process of families and caregivers with the goal of improving their ability to identify possible mental health issues as early as possible and to access appropriate help.

#### 3.1.4. Shortage of Linguistically and Culturally Appropriate Mental Health Services

If culturally and linguistically acceptable mental health services are unavailable, ethnic seniors with mental disorders often turn to traditional healers or religious leaders for help [21] or to ethnofocussed community and social agencies. However, generally there is poor service linkage and coordination between ethno-cultural social services and mainstream mental health programs. Hence, these ways of seeking help infrequently and inconsistently lead to effective mental health interventions [7].

The Wellness Centre has developed bridging strategies to ethnofocussed social agencies and community organizations by providing on-site regular mental health clinical consultation (in addition to education) to promote efficient and flexible paths of access to clinical care. In addition, direct referrals are accepted from social and community agencies, religious groups, traditional Chinese medical practitioners, and families and clients themselves.

Formal hiring policies reflect the goals of the centre. Members of the centre's multidisciplinary service team (a psychiatrist, social workers, a psychotherapist, a nurse practitioner, and a mental health promoter) are hired only from within the target community. All have relevant language and cultural skills and are trained by the program in culturally competent practices.

#### 3.1.5. Worries over Medication Side Effects and the Dominance of Drug Therapy

Deeply rooted culturally based conceptions of mental disturbance and concerns about an over reliance on drug therapy and psychotropic medication side-effects have often been voiced by ethnic groups [8, 21]. These conceptions and concerns lead to poor treatment compliance and diminished motivation to use mainstream services.

The Wellness Centre offers culturally relevant treatment options and self-help wellness programs to compliment evidence-based western psychiatric care and emphasizes the integration of culturally and language-appropriate psycho-education, psychotherapy, and supportive counselling.

#### 3.1.6. Underdetection of Psychogeriatric Problems at the Primary Care Level

Under-detection of psychogeriatric problems at the primary care level is a widely observed phenomenon [28]. To enhance early detection and to promote a seamless collaboration with primary care practitioners, the centre has worked closely with Chinese-speaking family doctors. A notable development was the establishment of a clinical education and shared-care physician group which has been mentored and supported by centre clinicians in providing shared care through regular group meetings to discuss clinical cases, continuing mental health education, and online consultations with Centre psychiatrists as needed. Referrals from this group of shared-care physicians accounted for one-third of all referrals from primary care to the Wellness Centre.

#### 3.1.7. Difficulties in Commuting to Specialized Services

The Wellness Centre is located in geographically accessible storefront units within a Chinese shopping plaza near the centre of the target community. The program provides transportation assistance and offers clinical outreach and home visits to homebound clients.

#### 3.1.8. Challenges Navigating the Complex Health System and Long Waiting Times

Long waiting periods for service and complicated referral procedures can often diminish clients' willingness to seek appropriate help and discourage referrals. The Wellness Centre created a simple and easy referral process that is user friendly and time efficient. The service inquiry or initiation process normally takes an average of 3–5 minutes and includes attention to the urgency of the requests and the need for expedited assessment and treatment.

The Centre has increased service capacity and reduced case-turnover times by forming partnerships of shared care with other agencies and enhancing their capacity to offer basic mental health care through education and onsite consultation.

## 4. Program Evaluation

Since its evolution into a psychogeriatric program in late 2005 to the end of 2009 (52 months), the Wellness Centre has provided wellness programs and community education, including anti-stigma initiative, to over 11,000 individuals (about 2,600 per year).

**Figure 2:**
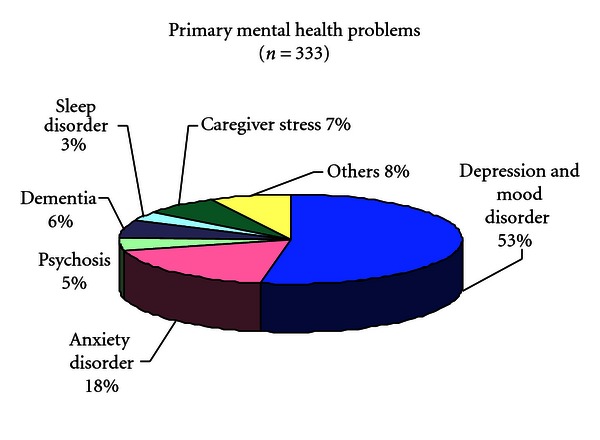
Primary mental health problems of clinical service clients.

During the same time period, Wellness Centre has focused on reaching vulnerable, underserved seniors who have not accessed services despite demonstrated needs. It has completed 333 comprehensive intakes of individuals and their families in the defined target group. Of those, 67% had no history of psychiatric care in Canada; 98% identified Chinese or a Chinese dialect as their preferred language and 70% spoke no or minimal English. Using the DSM-IV R Criteria, over half of these clients (53%) were diagnosed with mood disorders particularly depression; 18% suffered from anxiety disorders; 5% exhibited symptoms of psychosis, and another 6% had dementia The diagnostic or presenting complaint of clients is summarized in Figure 2.

**Figure 3:**
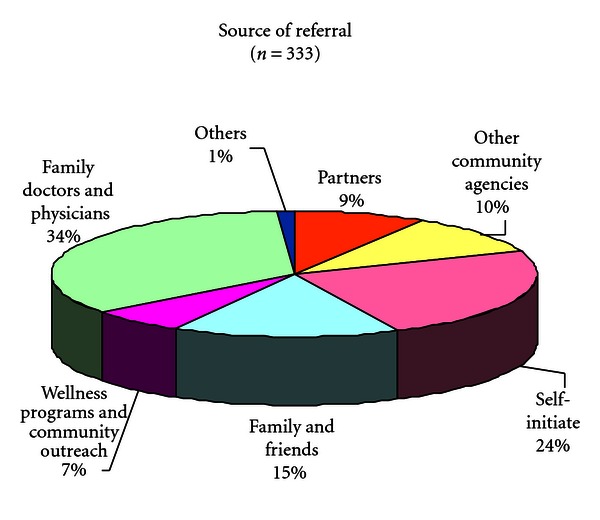
Source of Referrals.

**Figure 4:**
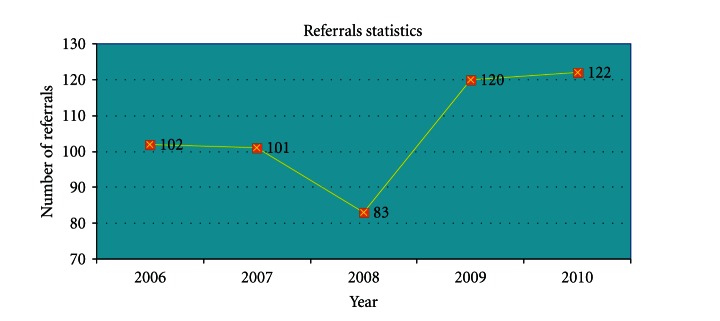
Number of Referrals Received by the Wellness Centre.

Referral statistics are an indicator of the success of the Centre in helping to improve community access and pathways to care of ethnocultural seniors (see Figure 3). Among cases referred by primary care physicians, 35% were from doctors that belong to the shared care network affiliated with the Wellness Centre. In 2009, there was a 46% increase in the number of referrals and requests for clinical services and a 47% increase in number of intake assessments completed (see Figure 4). The dip in referrals in 2008 may be related to more effective education of referral sources on the criteria for admission to the wellness program. In 2006-2007 some referrals were of patients younger than the age 55 cut-off, a residual effect of the prior general adult mandate of the program.

The Wellness Centre adopted the “Rate of Return” (ROR, or continuation rate) as one key measure of the effectiveness of the program. ROR is defined as continuation of treatment after 1 session [29, 30], excluding program-initiated discharge and is regarded as an indicator of success in patient engagement.

The ROR of the Wellness Centre is 93%. Of the 333 intakes, there were only 23 client-initiated withdrawals. Historically, the continuation rate among ethnic clients in mainstream programs in North America has been low compared to mainstream clients [29, 31]. The 93% ROR of the Wellness Centre is substantially better than the 48% return rate for Asian Americans reported by Sue [29], surpasses the highest ROR reported for community mental health programs of 88% (an outlier figure) reported by O'Sullivan et al. [30], and is comparable to the most optimistic figure (98%) specified for ethnicity-specific programs for Asian Americans in Takeuchi and colleagues' Los Angeles County study [31]. Considering that the geriatric clients served by the Wellness Centre may generally encounter greater barriers to remaining in treatment, such as transportation problems, level of acculturation, and reliance on family support, the Wellness Centre ROR may be a notable measure of success.

Table 1 summarizes the most recent convenience sample satisfaction survey of 85 clients and families. These figures acknowledge the Centre's culturally acceptable and accessible mental health services. Ninety-eight % were satisfied with the service received and would recommend the Wellness Centre to others in need of mental health services. All strategies were highly endorsed. The five most important were (1) language appropriateness, (2) a culture-friendly environment; (3) staff ethnicity reflective of client population; (4) cultural understanding and respect; (5) program location in the ethnic community.

## 5. Discussion

While there is some data in the literature, albeit sparse, on general adult ethnocultural and ethnospecific mental health programs, there is very little in the way of program description and data for ethnocultural seniors.

The Wellness Centre data suggest that systematically addressing barriers to accessing care for ethnocultural seniors may be effective in improving early identification of mental disorders, overcoming aspects of stigma, improving clinical outcomes through enhanced rates of retention, improving levels of patient and family satisfaction with psychiatric services, and successfully integrating community and mainstream resources.

While many or most jurisdictions recognize the importance of culturally competent mental health services, the viability and sustainability of a model of care such as that of the Wellness Centre are challenged by many factors: wavering political support for ethnocultural communities and the programs that serve them, competing priorities for ongoing funding, and the varying commitment of leaders of the affiliated health institutions to effective ethnocultural services. Moreover, the uncertainty of members of the community about the approach and need for services and the absence of good models to inform their development may lead to skepticism about the motivation and ability of mainstream institutions to undertake this work.

As the Wellness Centre model became more widely known there were heated debates among stakeholders about whether the wellness activities and the apparently broad-based admission criteria would steer the program away from its mental health funding mandates. Some argued that the “non-treatment” engagement-focused wellness activities would “contaminate” the purity of the mental health program and, therefore, obscure the image of the mental health service. Not pushing clients to accept psychiatric disease labels at the engagement stage was seen by some as an avoidance approach which might perpetuate community stigma against mental disorders. These criticisms highlight the importance of a well-articulated model such as the expanded wellness-dis-ease continuum, to ground the program and its rationales for its philosophy and function as a psychiatric and mental health service.

The success of a mainstream program is influenced by the strength of its partnerships. Successful and compatible partnerships allow mainstream programs to access a full spectrum of community-based support services that can complement the program's mental health intervention. Partnerships with agencies that are already embedded in and providing services for the target community help to ensure that services are culturally relevant and appropriate, that they are accepted by the community [32], and that they promote mainstream programs to access to local community resources and culturally relevant support programs [33]. Community partnerships also offer an effective venue for ethnic agencies to reflect on and advocate for the unmet needs and priorities of the target community [34]. Even when there is recognition of clear needs within communities, it is always necessary to address issues of possible overlapping mandates and competition for scarce resources. One strategy adopted by the Wellness Centre was to initiate proactive communication by leading and creating a network of ethnospecific mental health service providers. The network provided a forum to explore and define a coordinated collaborative care and service model respecting the strengths of all participants.

It is not uncommon for ethnocultural clinical programs to experience marginalization within mainstream health institutions. The program needs to be valued and regarded by the affiliated institution as an essential and integrated component of the core clinical services. By actively engaging in model development, program evaluation, research, publication, and professional education, the ethnocultural health program is more likely to be accepted, remain credible, and enhance the clinical and academic missions of the mainstream institution.

## 6. Conclusion

Clinical services for seniors in ethnocultural communities address a complex array of factors over and above the common factors affecting accessibility and acceptance of psychiatric and mental health services by seniors. Programs whose structure and function systematically address these factors may markedly improve early detection, acceptability of services and reduce key barriers to access to care for ethnocultural seniors.

## Figures and Tables

**Table 1:** Access-facilitating strategies rated in 2008 Client Satisfaction Survey.

Factors valued by respondents for accessing Wellness Centre's service (*n* = 62)					
Factors:	Strongly agree (%)	Agree (%)		Strongly agree (%)	Agree (%)
Speak my language*	58	40	Ethnic staff reflective of target ethnic population*	52	44
Understand and respect my culture*	50	45	Simple and flexible service access procedure	48	45
Offers nonpharmacological intervention, including psychotherapy	40	53	Comfortable and cultural-friendly environment*	55	44
Traditional and alternative care options	23	63	Located at ethnic community*	50	44
Involves family	40	50	Noninstitutional/nonmedicalized environment	39	47
Timely response	48	48	Nonstigmatized feeling	36	40

Out of 85 approached, 62 completed the survey, 6 declined, and 17 could not be reached. Of those who responded, over 98% were satisfied with the service received and would recommend the Wellness Centre to others in need of mental health services.

*The 5 strategies most valued by respondents.
